# Poly(Lactic-*co*-Glycolic Acid): Applications and Future Prospects for Periodontal Tissue Regeneration

**DOI:** 10.3390/polym9060189

**Published:** 2017-06-01

**Authors:** Xiaoyu Sun, Chun Xu, Gang Wu, Qingsong Ye, Changning Wang

**Affiliations:** 1 The State Key Laboratory Breeding Base of Basic Science of Stomatology (Hubei-MOST) and Key Laboratory of Oral Biomedicine Ministry of Education, School & Hospital of Stomatology, Wuhan University, 237 Luoyu Road, Wuhan 430079, China; Sunxyu198912@163.com; 2 Department of Oral Implantology and Prosthetic Dentistry, Academic Centre for Dentistry Amsterdam (ACTA), University of Amsterdam and Vrije Universiteit Amsterdam, Amsterdam Movement Sciences, Amsterdam 1081 HV, The Netherlands; g.wu@acta.nl; 3 Laboratory of Regenerative Dentistry, School of Dentistry, The University of Queensland, Brisbane 4006, Australia; c.xu3@uq.edu.au

**Keywords:** poly (lactic-*co*-glycolic acid), barrier membranes, bone grafts, drug delivery carriers, periodontal tissue regeneration

## Abstract

Periodontal tissue regeneration is the ultimate goal of the treatment for periodontitis-affected teeth. The success of regenerative modalities relies heavily on the utilization of appropriate biomaterials with specific properties. Poly (lactic-*co*-glycolic acid) (PLGA), a synthetic aliphatic polyester, has been actively investigated for periodontal therapy due to its favorable mechanical properties, tunable degradation rates, and high biocompatibility. Despite the attractive characteristics, certain constraints associated with PLGA, in terms of its hydrophobicity and limited bioactivity, have led to the introduction of modification strategies that aimed to improve the biological performance of the polymer. Here, we summarize the features of the polymer and update views on progress of its applications as barrier membranes, bone grafts, and drug delivery carriers, which indicate that PLGA can be a good candidate material in the field of periodontal regenerative medicine.

## 1. Introduction

**Figure 1:**
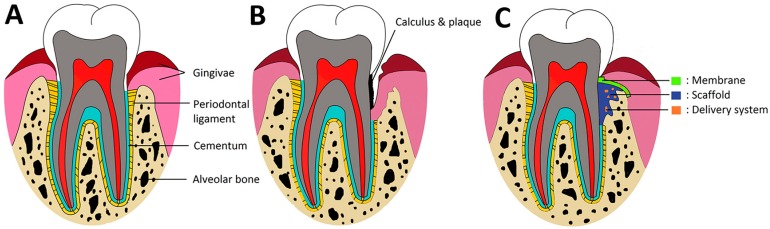
A schematic illustration illustrating the normal periodontal tissues (**A**); injured periodontium in periodontitis (**B**); and forms of PLGA applied for periodontal regeneration process (**C**). In (**C**), PLGA membranes can inhibit the early down-growth of gingival epithelium and connective tissues, allowing regenerative cells to repopulate the denuded root surface; PLGA based scaffolds can provide initial mechanical support and three-dimensional niches for neo-tissue formation; PLGA based delivery carriers can release the biological factors, and antimicrobial drugs to enhance periodontal regeneration.

Periodontal tissue regeneration is the ultimate goal of the treatment for teeth with periodontitis. Periodontitis is a highly prevalent inflammatory disorder that leads to early loss of tooth in adults and irreversible destruction of periodontium (the tooth-supporting apparatus). Take the United States of America as an example, as high as 46% of adults had periodontitis in 2009–2012, representing 64.7 million people [1]. The periodontium includes gingivae, periodontal ligament (PDL), cementum, and alveolar bone (Figure 1A) [2]. In the case of periodontitis, progressive loss of periodontium is involved, which mainly caused by microorganisms plaque (Figure 1B). Since the periodontium shows little tendency for self-repair once damaged [3,4], effective therapeutic interventions are needed to reconstruct the lost or injured tissues to gain their original structure and functions [5]. One approach, known as guided tissue regeneration (GTR), has drawn considerable attention to achieve complete periodontal tissue reconstruction [6,7]. It employs physical barrier membranes to exclude the unwanted gingival fibroblastic cells that proliferate at a faster rate than mesenchymal cells, allowing the slowly migrating osteogenic cells to repopulate into the defects [8]. Data from systematic reviews demonstrated that GTR yields advantageous outcomes in certain clinical scenarios, especially for degree II furcation lesions and intra-bony defects [9]. Another approach to achieve periodontal regeneration introduces bone grafting materials into periodontal defects, serving to accommodate and direct cells to grow, and contribute to mechanical stability of the defect sites [10,11]. Utilization of various types of bone grafts has resulted in some gain in clinical attachment levels and radiographic evidence of bone formation [12,13]. In addition, various drug delivery systems have also been applied to promote the periodontal regeneration where ability to control the release of bioactive molecules is important (Figure 1C) [14,15]. The success of periodontal regenerative modalities relies heavily on the utilization of appropriate biomaterials with specific properties.

PLGA, a synthetic copolymer of poly lactic acid (PLA) and poly glycolic acid (PGA), has been adopted in the production of various therapeutic devices including tissue grafts, surgical sutures, bone tissue engineering scaffolds, and drug carrier systems [16,17,18,19] due to their excellent biocompatibility, controllable biodegradability, tunable degradation rates, mechanical properties, and thermal processibility [20,21]. PLGA has been approved by the U.S. Food and Drug Administration (FDA) for human treatment and they can be easily prepared into versatile formulations, such as membranes, scaffolds, hydrogels, nanoparticles, microparticles, and sponges. All those properties make PLGA very attractive for periodontal regeneration (Figure 1C) and there are various commercialized products on the market. In addition, new research and strategies to improve the performance of PLGA for periodontal regeneration are springing up. In this review, the application of PLGA materials for periodontal tissue regeneration is described comprehensively. Firstly, the physiochemical properties, biocompatibility especially the pathway after implanting in body, and biodegradability of PLGA are introduced. Then, the comprehensive preclinical and clinical evaluation of PLGA as GTR membrane and bone grafting materials, and delivery systems are reviewed. Finally, recent progress and future perspectives of PLGA materials’ application in periodontal treatment are discussed.

## 2. Properties of PLGA Related to Periodontal Therapy

### 2.1. Physiochemical Properties

**Figure 2:**
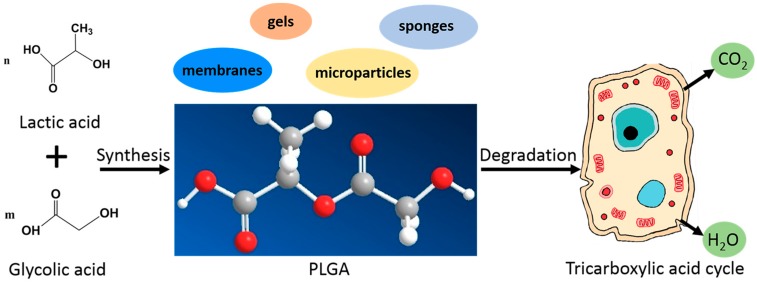
Synthesis, chemical structure, formulation and degradation of PLGA.

PLGA is a linear aliphatic copolymer obtained at different proportions between its constituent monomers, lactic acid (LA) and glycolic acid (GA) (Figure 2). It can be synthesized with any ratio of LA and GA, and molecular weights (*M*w) with a wide range from below 10,000 up to 200,000g/mol [22]. In addition, PLGA can be made in completely amorphous or highly crystalline forms. It has been reported that the polymer with less than 70% LA is amorphous in nature [23]. The amorphous form shows low mechanical strength, and is found to be suitable for drug release as it provides more even dispersion of a payload in the polymer matrix [19]. The crystalline form can be processed as surgical sutures and bone fixation vehicles with feasible mechanical strength.

PLGA is relatively hydrophobic, necessitating the use of organic solvents for formulation [18]. It is soluble in numerous organic solvents including tetrahydrofuran, chlorinated solvents, acetone or ethyl acetate [24]. Therefore, this polymer has been intensively utilized for drug delivery and to encapsulate both water-soluble and water-insoluble drugs [25]. The type of a drug (hydrophobic or hydrophilic) determines the preparation techniques of PLGA-based drug delivery system and solvents to be used in the processing procedures [21]. Furthermore, PLGA is available either with free carboxylic acids at the ends of polymeric backbone chain, or end-capped with alcohols [22]. The free carboxyl end-groups of the polymer can be used for chemical modifications to modulate its drug delivery properties considerably [26]. For example, the modification of PLGA with amino-bisphosphonate drug alendronate by covalent bonds provides a more sustained drug release kinetic which is beneficial for the treatment of metastatic bone diseases [27,28].Taken together, PLGA exhibits a large scale of physicochemical property diversities modulated by the variations in composite ratio, Mw, crystallinity, hydrophobicity, etc., which made them suitable for various biomedical devices [29,30].

### 2.2. Biocompatibility

All materials for medical devices that directly contact with human bodies should be biocompatible. They must not cause any systematic/local toxicity, and should perform with an appropriate host response. The biocompatibility of PLGA has been well investigated and documented. After in vivo implantation, the polymer can be gradually absorbed and replaced by fibrous connective tissue, bone tissue, and marrow tissues without causing tissue damage or other adverse effects [31]. When placed into dorsal skinfold chambers of rats, PLGA recruited vascular tissue in-growth, and were entirely traversed and penetrated by newly formed micro-vessels. The neovascularization degree and inflammatory response of the polymer were similar to that of the biological bone tissues [32]. A five-year clinical study also revealed that PLGA showed reliable biocompatibility and disintegration, where patients received PLGA plate osteosynthesis for maxillary and mandibular fracture reconstruction [33]. In view of its proven long-term clinical applications, PLGA is safe for biomedical applications. For response of periodontal tissue, in vitro assays showed that PLGA scaffolds promoted the proliferation of human periodontal ligament cells (PDLCs). In addition, PLGA also induced the osteogenic differentiation of PDLC as evidenced by the upregulated expression of osteogenic related proteins [34].

Furthermore, the degradation products of PLGA are LA and GA in an aqueous environment, which are endogenous chemicals. LA and GA enter the tricarboxylic acid cycle and is eliminated as carbon dioxide and water (Figure 2) [35,36]. GA can also be excreted through urine directly [36]. Thus, minimal systemic toxicity is associated with the use of the polymer for biomaterial applications [23]. Taken together, these findings provide significant evidence that PLGA is highly biocompatible and suitable for periodontal regeneration.

### 2.3. Biodegradability

The controlled degradability is a key factor for implantable biomaterials. The degrading materials will make room for the growth of new tissues, release incorporated bioactive molecules, and allow for the integration of any delivered cells into the surrounding tissues [37]. PLGA is biodegradable under physiological conditions, due to the presence of hydrolytically labile ester groups in its macromolecular backbone that can be broke down by cells [38]. The degradation time can be tailor-made, which varies from weeks to years to match the tissue formation timeframe by tuning the LA/GA ration, *M*w, etc.

The simplest ways to adjust the degradation rate of PLGA is to modulate its composition ratio of LA/GA [39]. Usually co-polymers with higher contents of GA are more hydrophilic and allow higher water permeability, resulting faster degradation rate. For example, PLGA50/50 with composition of 50% LA and 50% GA, exhibits a suitable degradation rate (approximately 1–2 months) [40], which was frequently applied for periodontal therapy. Except composition, Mw also has a profound effect on the biodegradation of PLGA, where generally polymers with higher Mw retained more structural integrity and exhibited longer degradation time [41].

In addition, the degradation process was also influenced by polymer end groups, degradation pH, temperature, etc. [21]. Since the hydrolysis of ester group in the backbone of PLGA triggered by water molecules, the hydrophobicity of the polymer end groups determines the water absorbing ability and the degradation rate. PLGA ended with acid group (hydrophilic) showed 2–3 folds faster degradation than those ended with ethyl or hexyl group (hydrophobic) [42]. PLGA capped with long carbon chain (lauryl alcohol, hydrophobic) showed a much slower degradation behavior and more sustained release of loaded drugs [43]. In addition to polymer end groups, an acidic medium accelerated the hydrolysis of PLGA as compared with a basic one [24,44], and the polymer experienced faster degradation at higher temperature [45].

## 3. Currently Commercially Available PLGA Products

PLGA is one of the most common and important polymers for medical applications because of its long-term clinical use and suitable properties as listed above. Thus far, there are 11 types of commercially available PLGA produces (Summarized in Table 1). They are formulated in different forms, including membranes (mesh), sponge, powers, gel, and suture with different LA/GA ratio. Their degradation time varies from a couple of weeks to 48 weeks. Some membranes products such as Vicryl^®^ and Resolut^®^ can remain integrity for at least 12 weeks, which meets the requirement for GTR or GBR.

## 4. Application of PLGA for Periodontal Regeneration

### 4.1. PLGA Barrier Membranes

The biodegradable and biocompatible nature of PLGA polymers, as well as the easy manipulate ability have made them good candidates for GTR membranes. The preclinical research using PLGA membranes for GTR therapy started from last century. In the 1990s, studies on primates with periodontal defects mimicking the situation of human beings, showed positive outcomes of accelerating the regeneration of periodontal tissues. In one study, where PLGA membranes were applied in the intrabony defects of rhesus monkeys, significantly higher amounts of new cementum, new bone formation, and connective tissue adhesion were achieved as compared to the flap operation only group (2.74 vs. 0.20 mm, 2.64 vs. 0.19 mm, 2.80 vs. 0.2 mm, respectively, details in Table 2). Similar results were observed in repairing Class II furcation defects of monkeys, and 250~350% more gain in the formation of new cementum, bone and connective tissue adhesion were observed using PLGA membranes [46] (Details in Table 2).

**Figure 3:**
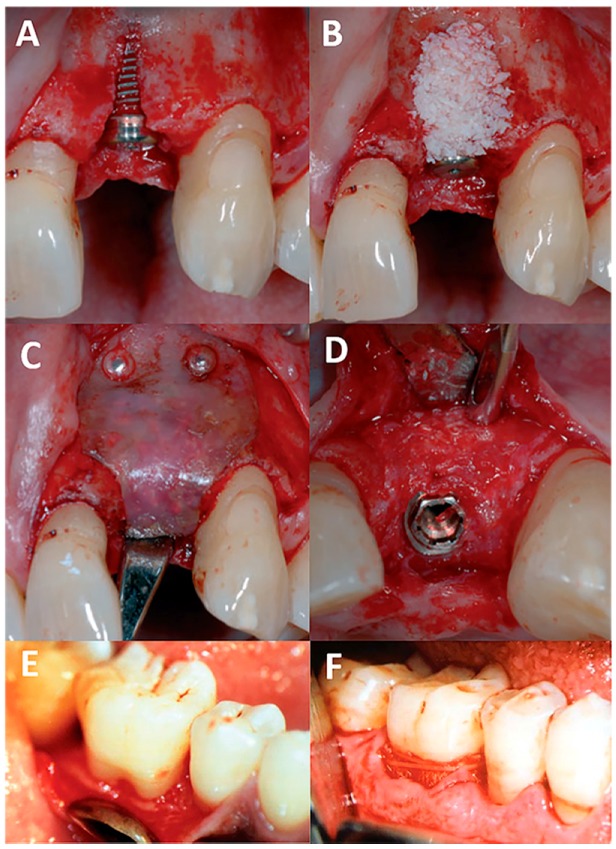
Clinical application of PLGA membranes for the treatment of intrabony defects. Adapted from [53,54] with permission. (**A**) shows the bone defect after implant placement and (**B**) shows the filling of bone substitute. A PLGA membrane was placed to cover the bone substitute and secured with two resorbable pins (**C**). After 6 months, the clinical examination after flap elevation shows the decrease of the defect height and integration of the implant (**D**) [54]. (**E**,**F**) show the clinical application PLGA membranes for Class II furcation defect, where reduction of furcation depth and gain in clinical attachment level can be observed [55].

In late 1990s, PLGA membranes were approved for clinical use in humans, mainly for the repairing of intrabony and class II furcation defects (Figure 3; Table 3). Most studies have shown that significantly greater clinical attachment level gain, less pocket probing depth and more bone fill were found in defects treated with PLGA membranes compared to flap operation only group. Tonetti et al. compared the efficacy between applying PLGA membranes and flap operation only in 154 patients with intrabony defects, and significantly higher clinical attachment level gain was found using PLGA membranes (3.04 vs. 2.18 mm, Table 2) [51]. Similar results were also observed by Aimetti et al. in 2005 for infrabony defects treatment, where average of 3.44 mm pocket probing depth reduction was achieved in PLGA group compared to that of 2.39 mm in flap operation ones. In addition to the improvements of clinical examination such as higher clinical attachments level, less pocket probing depth and less gingival margin recession, there was also more bone filling in the defect areas by radiologic examination in PLGA group, as compared to those with flap operation only (2.13 vs. 1.05 mm) [52]. For Class II furcation defects treatment, Balusubramanya et al. evaluated the clinical effects of PLGA membranes in 22 defects for six months, and concluded the use of resorbable periodontal mesh barriers resulted in reduction of furcation depth (1.54 mm) and gain in clinical attachment level (2.18 mm) [53].

The clinical performance of PLGA membranes over the other non-resorbable and resorbable membranes should also be critically assessed. Pretzl et al. conducted a 10 years study on 12 patients with 24 infrabony defects using PLGA and ePTFE (a typical non-resorbable membrane used in clinic) membranes. The results showed that significant gain in tissue attachment and bone formation were obtained in treatment with both membranes [59]. Study on the long-term stability suggested that the clinical benefits were maintained up to 10 years in most osseous defects. Though higher values in pocket probing depth reduction, clinical attachment level, and radiological bone filling were observed in PLGA membrane group, no statistic difference was found, which may due to the small sample size and big discrepancy among the subjects in this study. In another report, Bouchard et al. compared the PLGA membrane with ePTFE membrane in class II furcation defects, and concluded there was no statistical difference in pocket probing depth reduction, clinical attachment level gain and furcation depth reduction. For the comparison with other resorbable membranes such as collagen ones, PLGA showed similar results in periodontal regeneration for intrabony defects [57,58].

**Figure 4:**
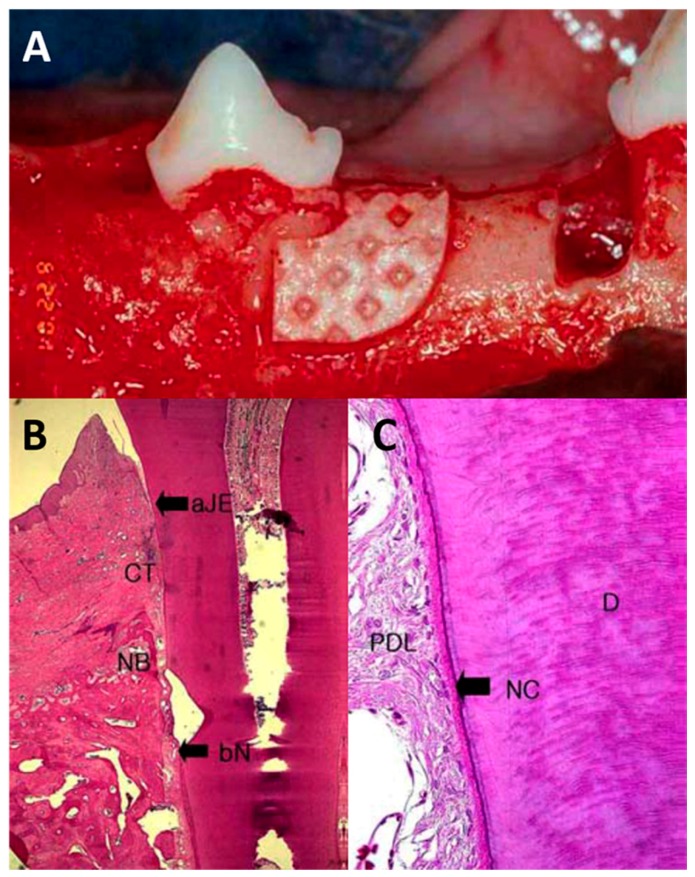
Photos of PLGA membranes for the treatment of intrabony defects in dogs. Adapted from [49] with permission. (**A**) shows the one-wall intrabony defects treated with PLGA membranes. (**B**) shows the defect treated with PLGA membrane demonstrated dense connective tissue formation, a moderate increase of new bone and new cementum, and (**C**) shows the perpendicular arrangement of periodontal ligament fibers in the areas of new bone and new cementum regeneration.

Furthermore, better results can achieved by using PLGA membranes loaded with antibacterial drugs, which are capable of inhibiting bacterial colonization, decreasing the risk of infection, and thereby contribute to increased gain of clinical attachment. Kim et al. [49] tested the therapeutic effect of PLGA membranes and tetracycline blended PLGA (TC-PLGA) on the preclinical one-wall intrabony defects of beagle dogs (Figure 4). Histological assessment showed significantly higher amounts of cementum and alveolar bone regeneration in the PLGA and TC-PLGA membrane group than those in the flap only group. The new cementum and bone amount were 1.48 ± 0.53 mm, 2.41 ± 0.21 mm and 2.97 ± 0.31 mm; 1.46 ± 0.68 mm, 2.39 ± 0.52 mm and 2.88 ± 0.66 mm, respectively, in the sham surgery control, PLGA membrane and TC-PLGA membrane group (details in Table 1). Though no significant difference with regards to the amount of regenerated new bone and cementum tissue was seen between the PLGA and TC-PLGA membrane group, TC-PLGA membranes showed additional gain of clinical periodontal attachment with less junctional epithelium migration, which may due to the antimicrobial properties of tetracycline during initial healing process.

### 4.2. PLGA-Based Bone Scaffolds

A vital treatment option for periodontitis involves the utilization of bone grafting materials that provide temporary mechanical support and porous architectures to guide cell migration and to initiate regenerative events [61,62]. PLGA has been investigated as bone scaffolds for periodontal defects, and prepared in different formulations such as powder, sponge and gel [63]. In one clinical study, Fishograft^®^ (PLGA with LA/GA ratio of 50/50) was applied to fill the infrabony defects. Significant reduction in probing depth, gain in clinical attachment level, and linear bone fill were observed [64]. Fishograft has also been applied for the augmentation of bone height through maxillary sinus lift procedures, and preservation of alveolar bone ridges following tooth extraction [65,66]. Furthermore, Fishograft has been proven to be optimum bone substitute for the regeneration of bone defects around immediate dental implant. The combination of autogenous bone graft and Fisiograft showed a slight superiority to autogenous bone graft alone.

### 4.3. PLGA for Periodontal Drug Delivery

Delivery of therapeutic agents such as growth factors and antibiotics has been proven to favor periodontal regeneration [67,68]. For an ideal drug delivery system for periodontal therapy, it should allow the impregnated agents to be released at intended rates and concentrations, and to linger at the desired sites for a sufficient period of time to recruit regenerative cells and stimulate tissue healing processes. PLGA, with various formulations such as hydrogels, membranes, microspheres, nanofibers, nanoparticles, and scaffolds, has been explored for the release of growth factors and antimicrobial drugs into the periodontium.

#### 4.3.1. Growth Factors Delivery

Various growth factors such as platelet-derived growth factor (PDGF), bone morphogenetic proteins, recombinant human growth/differentiation factor-5 (rhGDF-5), etc. have been used for periodontal therapy to enhance alveolar bone and PDL formation [4,69].The successful clinical experience of these factors relies mainly on the development of delivery vehicles or carriers that enable coordinated and orderly controlled release of biological agents [70,71].

A series of researches have investigated the regenerative effects of PLGA based growth factors delivery systems [72,73,74]. For example, rhGDF-5 loaded PLGA hydrogels have been applied to repair periodontal defects of dogs [75,76]. Results showed that the rhGDF-5 loaded hydrogels induced significantly more bone regeneration and cementum formation, and higher bone maturation than that in sham-surgery group. In addition, the hydrogels were injectable, avoiding the inconvenient surgical insertion of large implants [77]. In addition, an ideal growth factors delivery system has the ability to achieve spatiotemporal control which mimicking the physiological patterns of periodontal tissues [67]. Based on versatile degradation rates and high drug compatibility, PLGA with different structures has been designed to achieve precise release of several biomolecules in controlled and orchestrated profiles. Core–shell PLGA microspheres with double walls that encapsulate individual agents in either core or shell compartments have been fabricated to achieve consequently release of different drugs [78]. Chang et al. fabricated PLGA-Poly (d,l-lactide) microspheres with simvastatin and PDGF loaded in separate compartments to be released sequentially [79]. It is interesting to note that, by applying those microspheres, newly formed fibers were well-aligned and obliquely inserted onto the root surface. Furthermore, significant osteogenesis, bone maturation, and cementogenesis were observed [79]. Based on these results, it can be summarized that PLGA delivery carriers are promising vehicles for the combinational and sequential administration of growth factors, which accelerate periodontal regeneration.

#### 4.3.2. Antimicrobial Drug Delivery

Infection is one of the main causes of periodontal treatment failure [80]. Previous studies indicated that barrier devices impregnated with antibacterial drugs could decrease the risk of infection and contribute to increased gain of clinical attachment [81]. Tetracycline, a commonly used antibacterial agent, was successfully loaded into PLGA membranes [39]. The release profiles showed an initial burst phase in the first week followed by a sustained drug release over 14 days, which is desired for the needs of high antibiotic concentration in early phases but sufficient dose during the whole healing process. The loaded tetracycline did not affect the morphology of PDLCs that were seeded onto the membranes. Kim et al. [49] tested the release profile of tetracycline loaded PLGA membranes in vivo. Results showed that sustained tetracycline release with high concentration in the first 7 days, and higher amount of tissue attachment was observed compared to PLGA membranes only.

In addition to add the antibacterial drugs into PLGA directly, some other techniques such as surface coating or electrospinning are also reported to fabricate PLGA membranes with antimicrobial drugs. Gentile et al. developed a nano-layer coated PLGA membrane using layer-by-layer technique with a water-soluble antibiotic, metronidazole. They demonstrated the sustained release of metronidazole from functionalized PLGA membranes up to 25 days and the antibacterial abilities against Porphyromonas gingivalis, a keystone periodontal bacteria which can cause implant failure, without compromising biocompatibility of PLGA [82]. PLGA/gum tragacanth nanofibers fabricated by electrospinning method showed a core–shell structure and tetracycline was successfully loaded inside. The drug release rate can be effectively controlled with the core–shell nanofiber structure and the drug release can last for 75 days with small amount of burst release [83]. In vivo tests show PLGA devices with controlled release of antimicrobial drugs can significantly increase new bone formation in periodontal defects of dogs [84,85].

## 5. Future Directions of PLGA for Periodontal Regeneration

### 5.1. Surface Modification of PLGA Membranes for GTR

The traditional PLGA constructs act as passive barriers to exclude gingival epithelium and connective tissues so that undisturbed periodontal tissue regeneration occurs. To further improve the performance of the polymeric barriers, some investigators demonstrated the possibility of active roles for PLGA membranes by altering their surface topographies to selectively promote or inhibit cell proliferation and migration. The PLGA film whose surface cast with smooth, grooved, or rough topographies showed that such changes could guide and control cell activity. The grooved surface inhibited proliferation and migration of epithelial cells, and could be produced on the upper side that contacted the gingival epithelium. The rough surface promoted proliferation and directional migration of osteoblast cells, and thus could be placed on the lower side facing the bony defects, to guide and encourage the migration of osteoblasts into the defect areas [39]. Therefore, PLGA films with proper surface topographies on both sides have the possibility for enhancing tissue proliferation capacity and bone regeneration.

In additional to surface topographies, PLGA materials are easily to be combined with other materials to fabricate membranes with different surface composition. For example, a bi-layered PLGA/calcium phosphate membrane with outer layer of PLGA to hinder migration of epithelial cells and the inner layer of calcium phosphate to wick up blood clots and enhance osteoblast activity has been constructed [50]. Compared to flap-only control group, these membranes showed clearly better bone, cementum and PDL formation. PLGA membranes with appropriate surface topographies and composition may ensure better tissue regenerative outcomes than traditional devices.

### 5.2. Hydrophobicity Modification

Due to its hydrophobic nature, native PLGA has relatively low cell affinity. Various methods have been developed to modify the hydrophobicity based on the requirements of application [86]. The hydrophilicity of PLGA can be increased by raising the ratio of PGA in the copolymer or blended with other hydrophilic groups [87]. In addition, pre-wetted with solvents (e.g., ethanol, NaOH) has been reported to enhance the wettability of PLGA-based constructs to culture medium [88].

Plasma treatment represents another effective method to modulate the hydrophobicity and cell adhesion of PLGA. It can change the surface chemical composition of the polymer. Appropriate selection of the plasma source enables the introduction of diverse hydrophilic moieties, such as hydroxyl and peroxyl groups, onto the polymer surface to improve its hydrophilicity and enhance cell attachment [89]. Moreover, the plasma technique also introduces significant changes in topography. It may lead to the formation of peaks and valleys on the polymer surfaces. Different roughness elements can be obtained by adjusting the plasma etching time. The increased surface roughness can contribute to enhancing cell adhesion onto PLGA [90].

### 5.3. Improve Bioactivity

In recent years, focus has shifted from the use of the polymer in isolation to the application of functionalized PLGA-based composites [91,92]. PLGA lacks the natural recognition sites on its surface to promote cell attachment. Hence, extracellular matrix (ECM), ECM-like macromolecules, and functional peptide segments that act as biological cues for cell adherence are introduced to modulate the surface of the polymer [93]. Typically, PLGA are coated with bioactive agents, such as fibronectin, vitronectin, and collagen, to provide biomimetic interfaces between the polymer and cells [94,95]. The covalent linking of RGD (Arginine-glycine-aspartic acid) peptide sequences to biomaterials is a widely used technique in an attempt to mimic the ECM structure. The results positively indicated that cell attachment and proliferation were greatly promoted in the RGD modified PLGA mesh, as compared with that of the unmodified one. Moreover, the PLGA/RGD scaffolds resulted in better bone healing of rabbit mandibular defect [96]. In another study, Dai et al. [97] developed three-dimensional scaffolds, with collagen microsponges formed in the openings of porous PLGA knitted mesh. The composites retained mechanical properties from PLGA and biological properties from the collagen coatings. They offered advantages of high mechanical strength and enhanced cell seeding, and significantly facilitated tissue formation.

PLGA has relatively few bioactive groups, which limited its osteoconductive and osteoinductive properties, making it inadequate to induce mineralization. This problem can be solved by blending the polymer with ceramics, such as hydroxyapatite, calcium phosphate, and bioactive glasses [98,99]. Wang et al. [100] fabricated PLGA scaffolds whose surfaces were modified by nano-hydroxyapatite coating. The scanning electron microscopy image showed that biomimetic hierarchical nanostructures were found on the surface of the interior pores in the modified scaffolds, as compared to the relative smooth surface of the pure PLGA scaffolds. The modified scaffolds showed enhanced biological significance, as reflected by better biocompatibility, higher cell growth and proliferation rate, and superior regenerative capacity. Overall, the modification of PLGA and its combination with other biomaterials demonstrate the flexibility of this polymer and ensure its role as a vehicle in the fields of tissue regeneration and drug delivery.

### 5.4. PLGA Based Cell Engineering

To achieve predictable periodontal regeneration, it is necessary to utilize and recruit grafts not only function as space holders, but also have regenerative properties to enhance defect bridging [11]. This can be achieved by loading regenerative cells onto grafting materials. PLGA scaffolds are elastically compatible with human PDL and thus attracted particular interest for both cell seeding and cell transplantation [101,102]. Inanc et al. [103] established PDL tissue-like structures by seeding PDLCs onto nanoscaled PLGA mesh. Results indicated that the cells loaded on the meshes retained their viability, fibroblastic morphologies, phenotypical properties, and demonstrated up-regulated osteogenic marker expression. The constructs could be prepared with adjustable thicknesses. They were convenient to handle, and allowed for integration with host vasculature in the recipient sites. The resultant composites might be placed into periodontal wounds to promote tissue formation. Akita et al. [104] found that PLGA scaffolds that loaded with adipose-derived stromal cells could contribute to the reconstruction of periodontal fenestration defects in rats. The polymer scaffolds maintained spaces for new tissue growth. Histomorphometric analysis showed a significantly higher percentage of bone growth, thicker growth of PDL and cementum layers in the cell/PLGA groups as compared with polymer-only groups. It is also interesting to note that the alignment of PLGA fibers in the membranes can affect the growth behavior of PDLCs. Shang et al. [105] fabricated parallel and cross-aligned PLGA meshes, and found the aligned meshes allowed better cell attachment, proliferation, and directional migration as compared to that observed on randomly oriented meshes. Furthermore, the aligned constructs showed better structural stability and less shrinkage. Their mechanical strength was comparable to the strength of natural cancellous bone around the tooth root, and strong enough to maintain the defect space under pressure from gingival tissues and chewing forces.

Cementum allows the attachment of PDL to the denuded root surfaces, thus cementogenesis is of central importance to successful regeneration of periodontal tissues. PLGA scaffolds that loaded with cementoblasts had been tested to promote cementogenesis in animal models [106]. The scaffolds were fabricated by gas-foaming/particulate-leaching approach, and contained 95% porosity with pore sizes in the range of 250–425 µm. The results showed that the cells on the meshes retained their intrinsic properties. When placed into the dorsa of immunodeficient mice, the scaffolds induced remarkable mineral formation. New cementum was observed both within the pores of the implants and peripheral to the implants. Furthermore, PLGA constructs with rhGDF-5 and cementoblasts incorporated had proved to be powerful tools for periodontal regeneration. rhGDF-5 may unlock the latent regenerative potential of the periodontium by promoting cell recruitment and regulating cell proliferation and differentiation [77]. In all, the evidence suggested that PLGA scaffolds in combination with cementoblasts are viable for cementum engineering. The above researches demonstrated that PLGA scaffolds loaded with regenerative cells are promising bone grafts, and can be placed into the periodontal defects to reconstruct injured tissues.

## 6. Concluding Remarks

The remarkable advantages of PLGA such as excellent biocompatibility and tunable degradation properties ensure a wide range of opportunities for its application as barrier membranes, bone grafts, and delivery systems in periodontal regeneration, as demonstrated by adequate tissue formation in ample animal and clinical trials. However, pure PLGA exhibits poor hydrophilicity and suboptimal bioactivity, which presents a major bottleneck for its intensive application. To overcome this drawback, various modification strategies such as grafting bioactive groups onto the polymer surface or blending/copolymerizing the polymer with other materials have been introduced to endow the polymer with desired properties. Many efforts have been devoted to develop functionalized PLGA constructs with ability to deliver therapeutic drugs such as growth factors and antibiotics in a controlled manner, which has been proved to benefit the performance of PLGA devices such as PLGA barrier membranes for periodontal regeneration. In addition, PLGA based cell engineering also shows promise to reconstruct periodontal tissues. For future development, multifunctional PLGA devices with ability to elicit repair mechanisms of periodontal tissues and prevent unwanted fast migration of gingival fibroblastic cells in a biomimetic manner are desired achieve periodontal tissue regeneration which is the goal of periodontitis. We expect that this review will provide some insight into the design and development of PLGA constructs, allowing a selection of the most promising solutions to periodontal tissue regeneration.

## Figures and Tables

**Table 1:** Commercially available PLGA products for medical applications.

Trade Name	Manufacturer	Composition (ratio)	Form	Degradation time	Biocompatibility and tissue response	Mechanical properties	Features
Vicryl-Netz	Ethicon	PLA/PGA 10/90	Mesh	4–12 weeks	Inert, lack of tissue integration, no reactions in the surrounding tissues	Semicrystalline, relatively soft	Well adaptable, easy handling, elimination of membrane removal
Resolut	W.L. Gore	PGA/PLGA	Mesh	20–24 weeks	Good tissue integration, low inflammatory response in the surrounding tissues	Rigid, elastic	Retains its mechanical strength for 4 months to resist membrane collapse
Vicryl	Ethicon	PLA/PGA 8/92	Mesh	12 weeks	Good biocompatibility, limited inflammatory response	Semicrystalline, low elasticity, hold its tensile strength for 2–3 weeks in tissues	Easy to handle surgically
			Suture				
Polysorb	U.S. Surgical	PLA/PGA 80/20	Mesh	8–10 weeks	Minimal tissue reaction	High tensile strength, low elasticity	Easy handling
			Suture				
Dermagraft	ATS	PLA/PGA 10/90	Mesh	4 weeks	Good biocompatibility, limited immunological rejection, no inflammatory response	Great elasticity, porosity 95%, mechanical properties comparable to the native skin	Favorable for cell adherence
LactoSorb Screws and Plates	Walter Lorenz Surgical	PLA/PGA	Scaffold	48 weeks	Well tolerated, induced bone formation without causing adverse tissue responses	High tensile strength and stiffness, retains 70% of original strength at 8 weeks	Plates can be heated and molded to shape multiple times without compromising their mechanical strength
Biologically Quiet	Instrument Makar	PLA/PGA 85/15	Scaffold	24 weeks	No abnormal tissue reactions	Rigid, high mechanical strength, even stronger than the metal screws	Easy handling, avoidance of reoperation to remove the implants
Fisiograft	Ghimas S.p.A	PLA/PGA	Sponge	12–16 weeks	Biocompatible, totally absorbed in 3–4 months	Varies with different forms, mainly as bone filling materials	Osteoconductive, totally absorbable
			Power				
			Gel				
Lupron Depot	TAP	PLA/PGA 50/50	Microparticle	4 weeks	Minimal toxicity and minimal mechanical irritation to the surrounding tissues	Powders	Capable of delivering a sustained drug therapeutic level for 1 month
Zoladex	Astra-Zeneca	PLA/PLGA	Microparticle	4 weeks	Good biocompatibility, nontoxicity in most tissues	Powders	Monthly subcutaneous injection, increase patient compliance
ReGel	Macro-Med	PLGA–PEG–PLGA	Hydrogel	1–6 weeks	High biocompatibility	Some degree of flexibility	Compatible with tissues, ideally suited to deliver hydrophobic small molecules

**Table 2:** Application of PLGA membranes in periodontal defects treatment in big animal studies.

References	Animal types	Defect types	Length months	Treatment groups	New cementum (mm)	New bone (mm)	Connective tissue adhesion (mm)	Junctional epithelium extension (mm)
Hurzeler et al., 1997 [46]	rhesus monkeys	Intrabony defects	5	A flap operation only	0.20 ± 0.39	0.19 ± 0.37	0.20 ± 0.39	N
				PLGA membrane	2.74 ± 0.69 *	2.64 ± 0.74 *	2.80 ± 0.75 *	N
Hurzeler et al., 1997 [42]	rhesus monkeys	Class II furcation defects	5	A flap operation only	0.83 ± 0.19	1.14 ± 0.35	0.92 ± 0.26	N
				PLGA membrane	2.88 ± 0.63 *	2.78 ± 0.53 *	3.28 ± 0.55 *	N
Chang et al., 2000 [47]	dogs	Intrabony defects	3	PLGA membrane	4.03 ± 0.16	1.78 ± 0.22	N	0.92 ± 0.11
				PLGA membrane loaded with 25% doxycycline	3.89 ± 0.22	2.67 ± 0.30 *	N	1.04 ± 0.12
Kurtis et al., 2002 [48]	dogs	Intrabony defects	2	A flap operation only	0.97 ± 0.04	0.62 ± 0.07	0.96 ± 0.02	2.28 ± 0.06
				PLGA membrane	1.46 ± 0.09 *	2.01 ± 0.08 *	1.24 ± 0.02 *	1.16 ± 0.10 *
				PLGA membrane Loaded with metronidazole	1.53 ± 0.10 *	2.05 ± 0.08 *	1.20 ± 0.02 *	1.13 ± 0.10 *
Kim et al., 2007 [49]	dogs	Intrabony defects	2	A flap operation only	2.00 ± 0.70	1.46 ± 0.68	0.85 ± 0.43	N
				PLGA membrane	3.16 ± 0.37 *	2.39 ± 0.52 *	0.69 ± 0.17	N
				PLGA membraneLoaded with tetracycline	3.72 ± 0.53 *	2.88 ± 0.66 *	0.64 ± 0.10	N
Reis et al., 2011 [50]	dogs	Class II furcation defects	4	A flap operation only	N	trabeculaenumber = 1	3.80 ± 1.34	N
				PLGA membrane combined with CaP particles	N	trabeculaenumber ≈ 3 *	1.80 ± 0.44 *^,#^	N

N: These data were not available in this study. * Significant difference with other groups. ^#^ clinical attachment level instead of connect tissue adhesion was observed in this study.

**Table 3:** Clinical results of PLGA membranes for periodontal regeneration.

References	Patients number	Defect type (defect number)	Length (months)	Treatment groups	Pocket probing depth reduction (mm)	Clinical attachment level gain (mm)	Gingival margin recession change (mm)	Radiologic bone fill (mm)	Vertical/horizontal furcation depth reduction (mm)
Becker et al., 1996 [55]	50	class II furcation invasions/31	12	PLGA membrane	2.5 ± 1.4	2.1 ± 1.6	−0.4 ± 1.0	N	1.8 ± 2.0
Becker et al., 1996 [55]	50	Intrabony defects/30	12	PLGA membrane	4.0 ± 1.5	2.9 ± 2.0	1.2 ± 1.6	N	N
Bouchard et al., 1997 [56]	30	class II furcation defects/30	12	ePTFE membrane	1.8 ± 0.3	1.2 ± 0.3	N	N	2.7 ± 0.3
				PLGA membrane	2.1 ± 0.4	1.5 ± 0.5	N	N	2.5 ± 0.4
Tonetti et al., 1998 [51]	154	Infrabony defects/154	12	flap operation only	N	2.18 ± 1.46	N	N	N
				PLGA membrane	N	3.04 ± 1.64 *	N	N	N
Mattson et al., 1999 [57]	19	Infrabony defects/23	6	collagen membrane	1.66 ± 1.81	1.00 ± 1.82	0.66 ± 1.11	2.1 ± 2.18	N
				PLGA membrane	2.61 ± 1.75	2.01 ± 1.87	0.60 ± 0.99	1.67 ± 2.10	N
				PLGA membrane	1.8 ± 1.3	1.4 ± 1.2	N	N	N
Stavropoulos et al., 2004 [58]	28	Infrabony defects/28	12	PLGA membrane + Bio-Oss	4.0 ± 1.2	2.9 ± 2.3	1.1 ± 1.6	N	N
				collagen membrane + Bio-Oss	5.1 ± 1.7	3.9 ± 1.3	1.2 ± 0.8	N	N
Aimetti et al., 2005 [52]	18	Infrabony defects/36	12	flap operation only	2.39 ± 0.92	1.50 ± 0.99	0.89 ± 0.58	1.05 ± 0.94	N
				PLGA membrane	3.44 ± 0.78 *	2.89 ± 0.90 *	0.56 ± 0.92 *	2.13 ± 1.21 *	N
Pretzl et al., 2009 [59]	12	Infrabony defects/24	120	ePTFE membrane	2.4 ± 1.6	−1.7 ± 1.3	N	0.8 ± 0.6	N
				PLGA membrane	4.2 ± 2.5	0.2 ± 2.0	N	2.76 ± 1.70	N
Agarwal et al., 2012 [60]	12	Infrabony defects/16	6	bone allograft only	2.00 ± 0.19	1.38 ± 0.1	N	0.63 ± 0.26	N
				bone allograft with PLGA membrane	2.75 ± 0.37	1.50 ± 0.27	N	1.13 ± 0.23	1.37 ± 1.12
Balusubramanya et al., 2012 [53]	7	class II furcation defects/22	6	flap operation only	N	1.09 ± 0.94	N	N	1.54 ± 1.04 *
				PLGA membrane	N	2.18 ± 0.6 *	N	N	

N: These data were not available in this study. * Significant difference with other group.
